# Development and epidemiological investigation of a TaqMan-based multiplex real-time quantitative PCR assay for simultaneous detection of five bovine viruses (BVDV, AKAV, BNoV, BEV, and BCoV)

**DOI:** 10.3389/fvets.2026.1906498

**Published:** 2026-08-20

**Authors:** Hanjia Zhang, Yanbing Bai, Fan Zhang, Xuanyi Liu, Chunling Guo, Linyue Zhou, Ruichi Zhang, Sisi Wang, Qingfeng Meng, Hao Dong

**Affiliations:** 1 College of Life Sciences, Jilin Agricultural University, Changchun, Jilin, China; 2 Sanjiang Laboratory, Jilin Agricultural University, Changchun, China

**Keywords:** Akabane virus (AKAV), bovine coronavirus (BCoV), bovine enterovirus (BEV), bovine norovirus (BNoV), bovine viral diarrhea virus (BVDV), TaqMan-based multiplex real-time quantitative PCR

## Abstract

**Inroduction:**

Infectious diseases caused by bovine viral diarrhea virus (BVDV), Akabane virus (AKAV), bovine norovirus (BNoV), bovine enterovirus (BEV), and bovine coronavirus (BCoV) significantly threaten the cattle industry, resulting in substantial economic losses. These pathogens often present similar clinical signs, such as diarrhea, vomiting, and reproductive disorders in pregnant cattle, and frequent covert or mixed infections further complicate accurate diagnosis. Therefore, rapid, sensitive, and field‑deployable diagnostic methods are essential for effective disease surveillance and control in the cattle industry.

**Methods:**

In this study, we report for the first time the establishment of a TaqMan‑based real‑time quantitative PCR (qPCR) assay that enables simultaneous detection of these five bovine viruses. Multiple sequence alignment of conserved genomic regions was performed, and virus‑specific primers and probes were designed and optimized using Beacon Designer 7 software. Subsequently, a TaqMan‑based multiplex real‑time qPCR assay was established for simultaneous detection of BVDV, AKAV, BNoV, BEV, and BCoV. The established detection method was applied to 200 clinical samples collected from 10 farms in multiple regions of Jilin Province.

**Results:**

The results showed that the detection rates for BVDV, AKAV, BNoV, BEV, and BCoV were 33.50%, 0.50%, 4.50%, 7.50%, and 12.00%, respectively. Mixed infections were detected in 9 samples co‑infected with two of the five pathogens, with an overall mixed infection rate of 4.50%. Compared with conventional PCR, coincidence rates were 100% for BVDV, AKAV, BNoV, BEV, and BCoV.

**Discussion:**

These findings indicate that the TaqMan multiplex real‑time qPCR assay developed here demonstrates favorable specificity, sensitivity, and reproducibility. This assay enables efficient detection and surveillance of bovine viruses, offering a reliable technical tool for the diagnosis and control of corresponding viral diseases in cattle.

## 1 Introduction

With increasing stocking densities and herd sizes, the occurrence of infectious diseases in cattle has become more pronounced, posing a growing threat to herd health and productivity. Bovine viral diarrhea virus (BVDV), bovine coronavirus (BCoV), and Akabane virus (AKAV) are classified as Class III animal pathogens in China. Bovine norovirus (BNoV) and bovine enterovirus (BEV), although not included in this list, have progressively expanded their epidemic ranges with markedly increased detection rates. Jilin Province is a high-prevalence region for BVDV infection. A meta-analysis revealed that the BVDV pathogen prevalence in Jilin reached 26.30%, the highest among all provinces in China (1). The co-infection rate of BVDV and BEV in different areas of Jilin Province ranged from 3.42 to 14.71% (2). The BCoV antigen positive rate reached 21.10% (3), and the detection rate of BNoV in diarrheal samples reached 25.81% (4). Collectively, this epidemiological evidence indicates that these five viruses are prevalent in the cattle population of Jilin Province and frequently present as mixed infections. However, a method capable of rapidly and simultaneously differentiating these five pathogens has not yet been reported. Therefore, the aim of this study was to develop a multiplex real-time PCR assay for the simultaneous detection of BVDV, AKAV, BNoV, BEV, and BCoV.

BVDV, a member of the genus *Pestiviru*s within the family *Flaviviridae*, is a single-stranded positive-sense RNA virus (5). Three genotypes (BVDV-1, BVDV-2, and BVDV-3) have been identified to date (6). Previous studies have confirmed that BVDV can establish persistent infections by inducing host immune tolerance (7). This mechanism disrupts immune barriers and exacerbates the onset and progression of bovine respiratory disease complex, making BVDV one of the major immunosuppressive pathogens in cattle (8). AKAV, belonging to the genus *Orthobunyavirus* within the family *Bunyaviridae*, is a segmented, single-stranded negative-sense RNA virus (9). It is primarily transmitted by hematophagous arthropods, including *Culicoides* and mosquitoes, exhibiting distinct seasonal epidemiological patterns in tropical and temperate regions (10). Infection of pregnant cows with AKAV can cause abortion, stillbirth, fetal mummification, and arthrogryposis-hydranencephaly syndrome in newborn calves, seriously threatening herd reproductive performance (11). BNoV, classified in the genus *Norovirus* within the family *Caliciviridae*, is a single-stranded positive-sense RNA virus that primarily infects calves (12). It causes acute gastroenteritis characterized by mild to moderate diarrhea. Recent epidemiological surveys have indicated that BNoV frequently co-infects with other enteric viruses, and its detection rate in bovine diarrhea cases has steadily increased, highlighting its role as an important pathogen in calf diarrhea (13). BEV, belonging to the genus *Enterovirus* of the family *Picornaviridae*, is a single-stranded positive-sense RNA virus (14). It spreads horizontally among cattle mainly via the fecal–oral route and is widely distributed across diverse farming systems. Although BEV infections typically remain subclinical, existing evidence links BEV with calf diarrhea and respiratory symptoms (15). Frequent co-infections with BVDV, BCoV, and other pathogens substantially increase the complexity of clinical differential diagnosis and etiological tracing. BCoV, a member of the genus *Betacoronavirus* within the family *Coronaviridae*, is a single-stranded positive-sense RNA virus exhibiting dual tissue tropism for respiratory and enteric tracts (16). BCoV can infect respiratory mucosal epithelial cells, causing respiratory disease in calves and shipping fever in adult cattle (17). It can also invade intestinal epithelial cells, inducing severe diarrhea in calves. Consequently, BCoV is considered a key etiological agent of bovine respiratory disease complex and calf diarrhea (18).

All five of these pathogens are RNA viruses and exhibit considerable overlap in clinical manifestations. Infected cattle often present with systemic symptoms such as diarrhea, vomiting, and fever, whereas pregnant cows may experience abortion, stillbirth, and congenital infections (19). Therefore, accurate identification and differentiation based solely on clinical signs are challenging. Additionally, these viruses frequently persist in cattle herds in subclinical or persistent states, and mixed infections are common (20). These factors further complicate clinical diagnosis, outbreak tracing, and precise disease control. Traditional single-pathogen detection methods have inherent limitations, including low efficiency, high cost, and susceptibility to false-negative results, rendering them insufficient for current demands of rapid screening, early warning, and precise intervention (21). Consequently, the development of a highly efficient, specific, multiplex real-time quantitative PCR (qPCR) assay for simultaneous detection of these five major bovine RNA viruses, BVDV, AKAV, BNoV, BEV, and BCoV, holds significant theoretical and practical value. Such an assay can enhance laboratory-based surveillance capabilities and enable early and accurate differential diagnosis.

**Figure 1:**
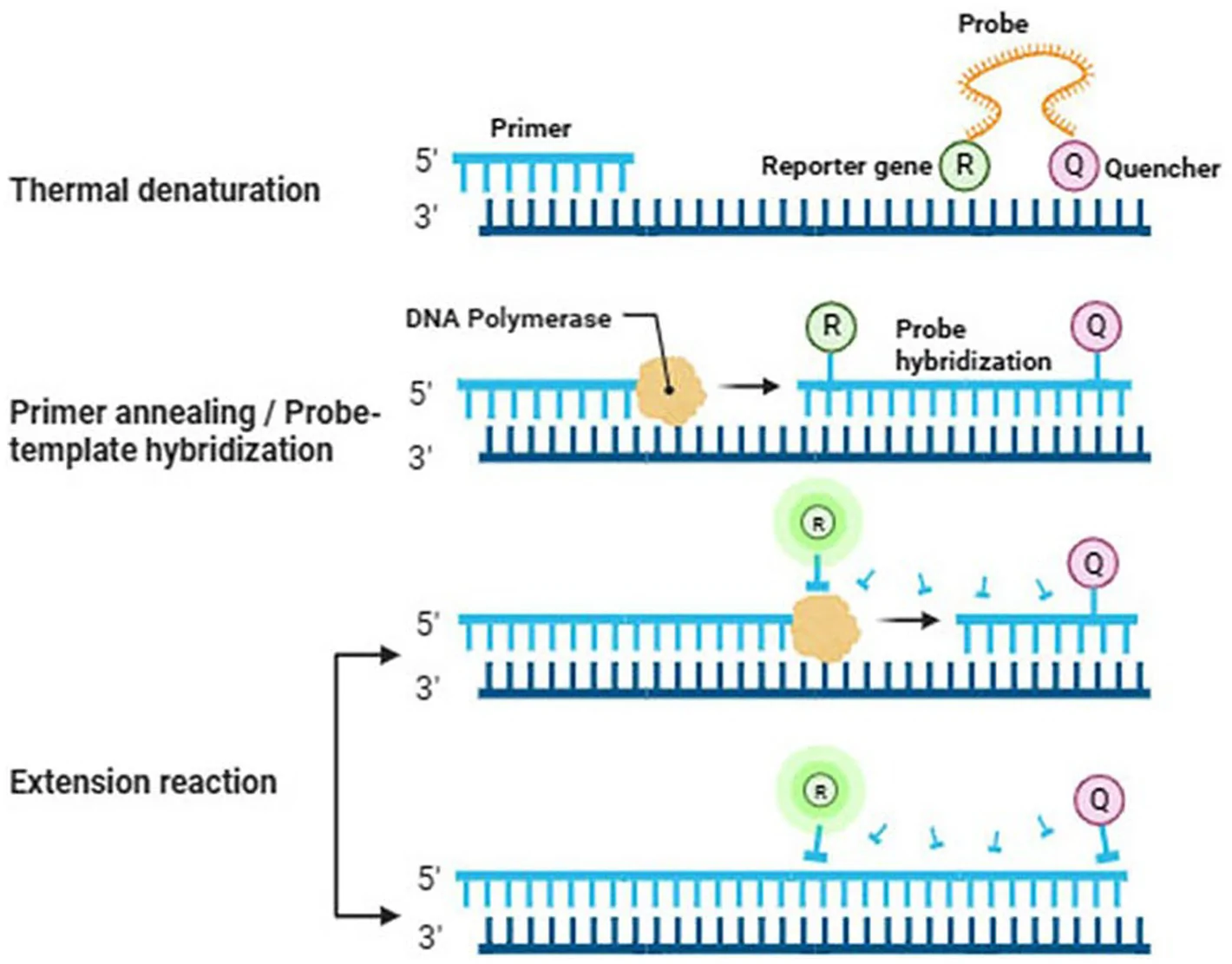
The core mechanism of the TaqMan probe assay relies on the 5′ → 3′ exonuclease activity of Taq DNA polymerase, which cleaves the probe to generate a fluorescent signal. When the probe remains intact, the fluorescence emitted by the reporter group is absorbed by the quencher group, resulting in no detectable fluorescence signal. During the PCR amplification reaction, the 5′ → 3′ exonuclease activity of Taq polymerase hydrolyzes and cleaves the probe, physically separating the reporter group from the quencher group and thereby producing a measurable fluorescent signal.

The core mechanism of the TaqMan probe-based real-time qPCR assay relies on the 5′ → 3′ exonuclease activity of Taq DNA polymerase (22). During PCR amplification, the enzyme specifically cleaves the probe bound to the target sequence (23), thereby releasing a fluorescent signal that allows for real-time quantitative detection (Figure 1). Due to its exceptional specificity, high sensitivity, and quantitative capability, TaqMan probe technology is widely adopted across various fields, including medical diagnostics, molecular biology research, veterinary pathogen detection, and quantitative analysis of genetically modified foods and agricultural products (24). It has thus become an essential molecular diagnostic technique. In veterinary pathogen detection, Yu et al. developed a multiplex RT-qPCR assay based on TaqMan probes for simultaneous detection of three goose-origin viruses (GoAstV, GPV, and GoCV) (25). In their study, the detection limits for the three viruses reached 10 copies/μL in the single-plex real-time RT-qPCR assay, whereas multiplex assay limits were approximately 100 copies/μL. The authors noted that competitive interactions among primers, probes, nucleic acid templates, and reaction components in multiplex systems typically result in slightly reduced sensitivity compared with single-plex assays (26). This represents a key technical challenge in optimizing multiplex molecular detection methods.

In this study, specific TaqMan probes and primers targeting conserved genomic regions of BVDV, AKAV, BNoV, BEV, and BCoV were designed. A multiplex real-time fluorescence qPCR assay capable of simultaneously identifying these five viruses was successfully established. Standard positive plasmids (pUC57-BVDV-5′UTR, pUC57-AKAV-S, pUC57-BNoV-RdRp, pUC57-BEV-5′UTR, and pUC57-BCoV-N) were constructed, and key performance characteristics including specificity, sensitivity, and reproducibility were systematically evaluated. The developed method is suitable for implementation in veterinary diagnostic laboratories and for rapid on-site detection in intensive farming operations, thereby providing a reliable technical foundation for early warning, accurate diagnosis, and comprehensive control of these five viral infections in cattle herds.

## 2 Materials and methods

### 2.1 Primer and probe design

Recombinant plasmids containing the 5′UTR gene of BVDV, S gene of AKAV, RdRp gene of BNoV, 5′UTR gene of BEV, and N gene of BCoV were constructed and stored in our laboratory.

Based on published sequences from the GenBank database (accession numbers KF925509.1, NC_009896.1, MK159169, LC150009.1, and KT318096.1), specific primers and probes targeting these five genes were designed using Beacon Designer 7 software (Table 1). All primers and probes were synthesized by Shanghai Sangon Biological (Shanghai, China). The synthesized primers and probes were centrifuged, diluted to 10 μM with ddH_2_O, and stored at −20 °C until further use.

**Table 1:** Probe and primer sequences.

Primer name	Primer sequences (5′ to 3′)	Length (bp)	Reaction volume (10 μmol/L)
BVDV-F	CAGGGTAGTCGTCAGTGGTT	115	0.3 μL (0.15 μM)
BVDV-R	TTTAGTAGCAATGCAGTGGG		0.3 μL (0.15 μM)
BVDV-P	FAM-TGAGTACAGGGTAGTCGTCAGTGGTTC-BHQ1		0.4 μL (0.20 μM)
AKAV-F	TTACATAAGACGCCACAACC	171	0.3 μL (0.15 μM)
AKAV-R	CAGAAACATCTCAGCACCAG		0.3 μL (0.15 μM)
AKAV-P	TET-ATCTAAGTTGGACGCAGTCAAGGCTG-BHQ1		0.4 μL (0.20 μM)
BNoV-F	GGCAAACCACGCAAACAACA	102	0.3 μL (0.15 μM)
BNoV-R	GACTACCTTCCCACAGTGACAGA		0.3 μL (0.15 μM)
BNoV-P	VIC-TTTCGGAAGTCACAAACCTTGACCCG-BHQ1		0.6 μL (0.30 μM)
BEV-F	TTTCTGCGGATTGGTATGAG	160	0.3 μL (0.15 μM)
BEV-R	TGAGTTCTGTGGTCCTGTTGA		0.3 μL (0.15 μM)
BEV-P	CY5-CAACTTACGCATTACGACGCAGCAAC-BHQ2		0.9 μL (0.45 μM)
BCoV-F	GCTATTCCGACTAGGTTTCC	204	0.5 μL (0.25 μM)
BCoV-R	CATTCTTCTGACCACGCTGA		0.5 μL (0.25 μM)
BCoV-P	NED-TGATAATATAAGTGTTGCAGCGCCCAA-BHQ2		0.5 μL (0.25 μM)

### 2.2 Preparation of standard plasmids

Sequences of the 5′UTR gene of BVDV, S gene of AKAV, RdRp gene of BNoV, 5′UTR gene of BEV, and N gene of BCoV were retrieved from the NCBI database. Multiple sequence alignments of the five viral gene sequences were performed, and highly conserved regions were selected as targets for recombinant plasmid construction. Following synthesis, single colonies were inoculated into LB liquid medium containing ampicillin and cultured at 37 °C with shaking for 12–15 h. Subsequently, recombinant plasmids were extracted using the OMEGA Plasmid Mini Kit (Omega Bio-tek, Norcross, GA, USA) according to the manufacturer’s instructions. Plasmid concentrations were measured and converted to copy numbers using the template copy number calculation formula, and stored at −20 °C for further use. Before the experiment, the specificity of the designed primers and probes was validated. Amplification was performed in a QuantStudio 3 real-time PCR system using the designed primer-probe combinations, with standard positive plasmids (pUC57-BVDV-5′UTR, pUC57-AKAV-S, pUC57-BNoV-RdRp, pUC57-BEV-5′UTR, and pUC57-BCoV-N) as templates (2 × ZAPA3G Probe Direct qPCR Mix, Beijing Zhongshi Tongchuang Biotechnology Co., Ltd., Beijing, China) (Tables 2, 3).

**Table 2:** Amplification reaction system and amplification reaction condition.

Reagents	Volume (μL)	Final concentration
2 × ZAPA3G Probe Direct qPCR Mix	10	1×
PCR Forward Primer (10 μM)	0.4	0.2 μM
PCR Reverse Primer (10 μM)	0.4	0.2 μM
Probe (10 μM)	0.8	0.4 μM
DNA	2	--
ddH_2_O	6.4	--
Total	20	--

**Table 3:** Amplification reaction condition.

Step	Temperature (°C)	Time	Cycles
Contamination digestion	37	2 min	1
Initial denaturation	95	5 min	1
Denaturation	95	10 s	40
Annealing/Extension	60	30 s	

### 2.3 Optimization of reaction conditions

Under initial reaction conditions, combinations of primers and probes were systematically optimized using a matrix method (Nanjing Vazyme Biotech Co., Ltd., Nanjing, China). During optimization, primer volumes (10 μM) were tested at 0.3, 0.4, 0.5, 0.6, 0.7, 0.8, 0.9, and 1.0 μL, with corresponding adjustments of probe volumes (10 μM) across the same gradient. The annealing temperature was optimized over a range of 55 °C to 60 °C in increments of 1 °C (55, 56, 57, 58, 59, and 60 °C). Based on optimal primer-probe concentrations and annealing temperature, a TaqMan multiplex PCR assay was successfully established.

### 2.4 Establishing standard curves

Standard plasmids for BVDV, AKAV, BNoV, BEV, and BCoV were prepared by 10-fold serial dilution, ranging from 10^7^ to 10^3^ copies/μL. Using ddH_2_O as a negative control, TaqMan real-time fluorescent qPCR amplification was conducted. Results were analyzed by plotting cycle threshold (Ct) values on the Y-axis against template copy numbers on the X-axis to generate standard curves.

### 2.5 Robustness test

To evaluate the anti-interference performance of the established multiplex real-time fluorescence qPCR assay, standard positive plasmids were used as positive controls, and ddH_2_O was used as the negative control. Positive nucleic acids of IBRV, BRV, BPV, BToV, and BRSV were spiked into the reaction system to assess assay specificity against non-target pathogens.

### 2.6 Specificity test

The specificity of the multiplex real-time fluorescent qPCR assay was evaluated using positive nucleic acids of BVDV, AKAV, BNoV, BEV, and BCoV, along with positive nucleic acids from infectious bovine rhinotracheitis virus (IBRV), bovine rotavirus (BRV), bovine papillomavirus (BPV), bovine torovirus (BToV), and bovine respiratory syncytial virus (BRSV). The reactions were performed under optimized conditions, with three replicates per group, and included a negative control.

### 2.7 Sensitivity test

A 10-fold serial dilution of standard plasmids for BVDV, AKAV, BNoV, BEV, and BCoV was prepared, with concentrations ranging from 10^10^ to 10^1^ copies/μL. Sterile double-distilled water was used as the negative control. A TaqMan real-time fluorescent qPCR assay was performed to evaluate assay sensitivity and determine the minimum detectable copy number for each pathogen.

### 2.8 Repeatability test

To evaluate the reproducibility of the assay, three positive templates with known concentration gradients were selected. For intra-assay repeatability, these templates were tested within the same experimental batch, with three replicates per concentration. For inter-assay repeatability, the same templates were tested at different time points, with three replicates per sample in each batch.

Using a 10-fold dilution series, the concentration gradients of standard plasmids for BVDV, AKAV, BNoV, BEV, and BCoV were set from 10^6^ to 10^4^ copies/μL. Both intra-assay and inter-assay experiments were performed, and the coefficient of variation (CV) was calculated to assess assay reproducibility (Table 4).

**Table 4:** Optimal primers and probes reaction system.

Primer name	Volume (μL)
BVDV-F	0.3
BVDV-R	0.3
BVDV-P	0.4
AKAV-F	0.3
AKAV-R	0.3
AKAV-P	0.4
BNoV-F	0.3
BNoV-R	0.3
BNoV-P	0.6
BEV-F	0.3
BEV-R	0.3
BEV-P	0.9
BCoV-F	0.5
BCoV-R	0.5
BCoV-P	0.5
DNA	3
2 × T5 Premix Plus (Probe)	25
ddH2O	14.3
Total	50

### 2.9 Sample testing

A total of 200 clinical samples (feces, nasal swabs, throat swabs, and blood samples) were collected from representative regions of Jilin Province, including large-scale dairy farms, individual livestock farms, and slaughterhouses. Extracted nucleic acids were used as templates for amplification using the established TaqMan multiplex real-time PCR assay (Table 4). Results were analyzed using the QuantStudio 3 PCR system. Subsequently, results were compared with those obtained by conventional PCR (Table 5).

**Table 5:** PCR reaction system.

Reagents	Volume (μL)
2 × Fast Taq PCR MasterMix	10
PCR Forward Primer (10 μM)	0.5
PCR Reverse Primer (10 μM)	0.5
DNA	2
ddH_2_O	7
Total	20

Sample Preparation Methods: For fecal samples, sterile phosphate-buffered saline (PBS) was added to the samples in centrifuge tubes, which were then vortexed until the solid material was thoroughly dissolved. After centrifugation at 12,000 rpm for 10 min, the supernatant was collected and filtered twice through 0.45-μm membranes. The filtrate was stored at −80 °C until further processing. For swab samples (nasal and throat swabs), the swabs were immersed in 1.5 mL of transport medium containing 1% antibiotics and subjected to three freeze–thaw cycles, followed by centrifugation at 4,000 rpm for 10 min. The resulting supernatant was collected and stored at −80 °C. For blood samples, the collected blood was allowed to clot at room temperature for 2–4 h, after which the serum was separated by centrifugation at 3,000 rpm for 5–10 min; the serum was then collected and stored at −80 °C. Total RNA was extracted from all processed samples using a viral RNA extraction kit, and reverse transcription was performed to obtain cDNA. Both the extracted RNA and the resulting cDNA were stored at −80 °C for subsequent analyses.

## 3 Results

### 3.1 Preparation of positive plasmids

**Figure 2:**
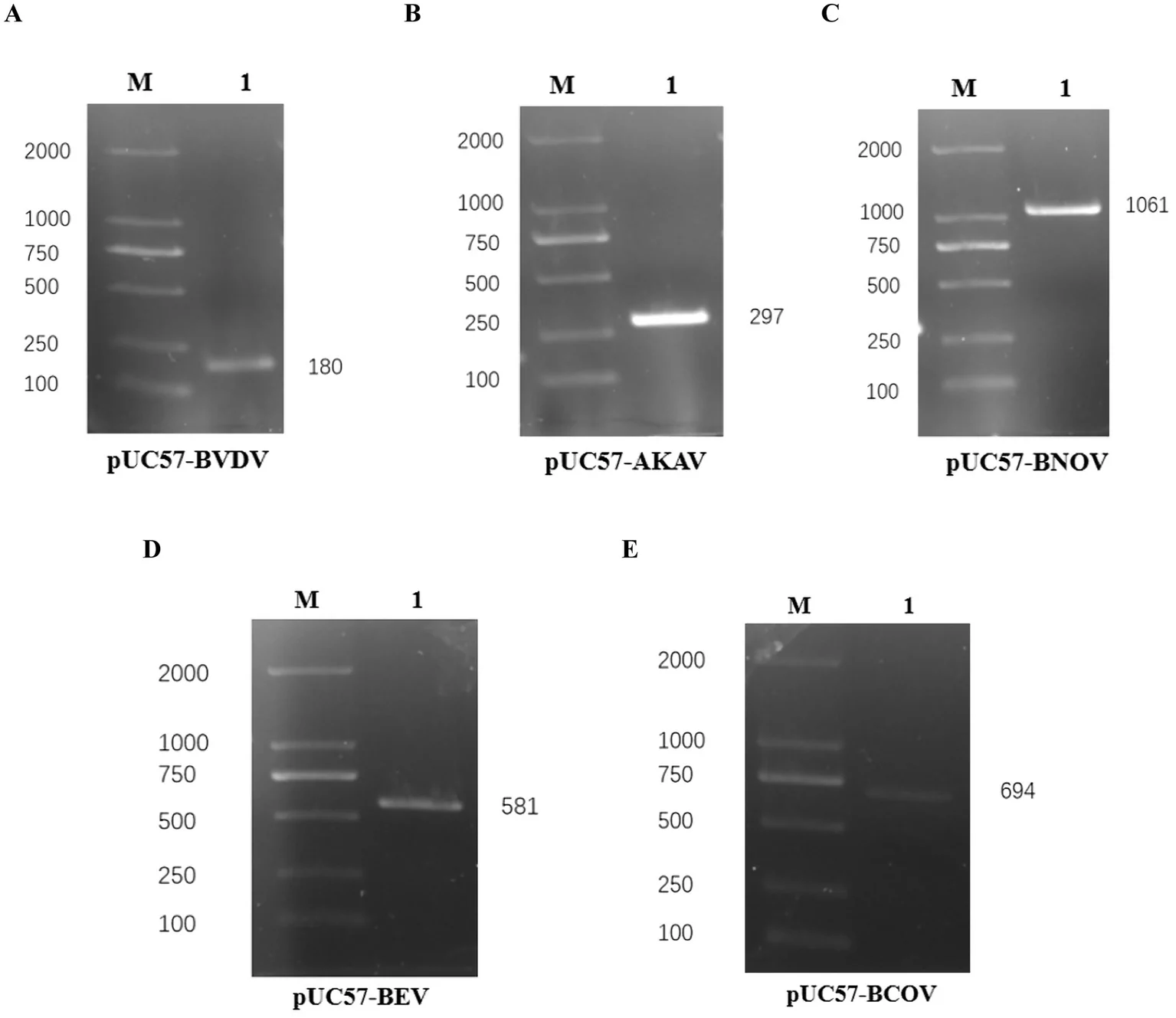
Standard positive plasmid construction results. **(A)**, pUC57-BVDV-5’UTR (180 bp); **(B)**, pUC57-AKAV-S (297 bp); **(C)**, pUC57-BNoV-RdRp (1,061 bp); **(D)**, pUC57-BEV-5’UTR (581 bp); **(E)**, pUC57-BCoV-N (694 bp). (M: DNA marker, 1: pUC57-virus).

Full-length primers were used to amplify recombinant plasmids pUC57-BVDV-5′UTR, pUC57-AKAV-S, pUC57-BNoV-RdRp, pUC57-BEV-5′UTR, and pUC57-BCoV-N. Analysis by 1% agarose gel electrophoresis showed that the target band sizes were consistent with the expected sizes (Figure 2). The recombinant plasmids were sequenced and compared with reference sequences in the NCBI database using BLAST, showing 99% sequence homology, which confirmed successful plasmid construction.

### 3.2 Preparation of standard plasmids

Standard plasmid concentrations were determined using a NanoDrop 2000 ultramicrospectrophotometer. The concentration of pUC57-BVDV-5′UTR was 123.9 ng/μL with an A260/280 ratio of 1.82; pUC57-AKAV-S was 151.6 ng/μL with an A260/280 ratio of 1.85; pUC57-BNoV-RdRp was 240.4 ng/μL with an A260/280 ratio of 1.85; pUC57-BEV-5′UTR was 236.2 ng/μL with an A260/280 ratio of 1.87; and pUC57-BCoV-N was 243.9 ng/μL with an A260/280 ratio of 1.87.

Plasmid copy numbers were calculated using the following formula: the copy number of the BVDV plasmid was 3.77 × 10^10^ copies/μL, AKAV plasmid was 3.88 × 10^10^ copies/μL, BNoV plasmid was 5.20 × 10^10^ copies/μL, BEV plasmid was 6.10 × 10^10^ copies/μL, and BCoV plasmid was 5.48 × 10^10^ copies/μL. Each plasmid underwent 10-fold serial dilutions, was aliquoted, and stored at −20 °C for further use.

Calculation method for copy counts:

$\text{Copy number}\;(\text{copies}/\mathrm{\mu L})=\frac{\left(6.02\times 1023)\times (\mathrm{ng}/\mathrm{\mu L}\times 10^{-9}\right)}{\left(\text{DNAlength}\times 660\right)}$

### 3.3 Reaction system optimization

Reaction conditions were optimized using a matrix-based approach. Optimized results for BVDV, AKAV, BNoV, BEV, and BCoV are presented in Table 6, respectively. Data shown represent the mean Ct values from triplicate wells. Conditions yielding lower mean Ct values were selected as optimal reaction parameters. No significant differences in reaction outcomes were observed after optimizing annealing temperatures; thus, 60 °C was chosen as the optimal annealing temperature. The final optimized reaction system is shown in Table 4.

**Table 6:** Optimization results. (The values marked in red represent the optimal reaction parameters.)

Samples	Primer								
	Probe	0.3	0.4	0.5	0.6	0.7	0.8	0.9	1.0
BVDV	0.3	16.047	22.026	16.941	22.210	23.107	25.378	24.733	24.340
	0.4	15.644	16.099	24.861	19.589	20.099	23.838	19.825	24.804
	0.5	15.920	19.853	18.058	28.618	24.057	26.584	23.847	23.688
	0.6	19.161	17.733	20.859	26.468	24.704	20.668	22.315	24.578
	0.7	23.833	26.821	21.811	18.585	22.438	22.287	22.986	23.462
	0.8	16.127	17.834	19.936	23.271	15.974	25.016	37.711	24.940
	0.9	17.732	24.989	23.222	22.549	28.293	20.488	20.087	26.400
	1.0	15.965	18.953	21.385	22.709	24.648	22.649	21.904	25.643
AKAV	0.3	13.724	20.699	22.055	21.913	23.047	23.608	24.058	27.137
	0.4	12.688	20.330	18.809	22.730	23.943	20.889	25.625	21.849
	0.5	17.621	19.914	25.592	26.489	18.796	22.998	26.390	13.750
	0.6	19.156	25.516	23.949	19.715	17.769	26.253	27.536	28.083
	0.7	26.385	23.753	26.021	23.774	27.600	24.451	25.234	25.075
	0.8	24.908	21.053	23.846	23.599	25.751	20.823	26.856	24.644
	0.9	13.727	19.607	27.775	24.051	27.488	27.462	26.439	26.487
	1.0	23.915	28.179	22.984	22.022	27.836	23.057	25.217	26.646
BNoV	0.3	11.452	20.774	21.363	19.619	17.827	20.203	23.595	25.404
	0.4	13.729	17.723	20.427	19.280	21.124	18.857	23.391	24.947
	0.5	11.019	19.688	21.168	11.709	18.127	24.733	23.008	22.698
	0.6	10.386	19.060	12.992	16.022	21.937	23.646	24.924	24.935
	0.7	12.407	18.526	21.769	19.193	23.802	21.001	22.762	23.359
	0.8	14.037	17.564	15.059	20.140	13.569	22.077	22.128	24.290
	0.9	20.024	23.858	22.317	11.513	13.024	23.963	22.200	23.121
	1.0	23.763	22.575	12.231	22.722	24.907	24.898	20.288	26.590
BEV	0.3	22.655	23.565	24.544	23.786	24.786	25.656	27.545	24.962
	0.4	23.350	22.896	25.137	25.875	24.436	28.455	24.786	23.765
	0.5	24.659	28.543	27.754	29.655	26.554	26.787	27.357	28.350
	0.6	23.762	25.752	25.895	25.753	27.876	28.652	25.766	29.762
	0.7	24.872	24.984	25.347	25.359	27.760	26.259	27.353	26.866
	0.8	23.654	27.225	25.327	26.632	25.670	24.365	23.634	26.350
	0.9	22.355	25.544	24.435	25.330	25.463	28.532	27.896	28.658
	1.0	24.914	25.214	27.354	26.546	25.667	28.453	27.356	30.436
BCoV	0.3	13.864	15.754	12.870	13.000	14.850	13.763	13.963	17.860
	0.4	15.649	20.765	14.762	17.971	17.537	16.352	18.530	21.576
	0.5	18.643	15.870	11.036	12.860	12.654	11.994	12.042	12.340
	0.6	16.876	12.858	14.895	14.168	12.667	15.876	19.665	21.654
	0.7	15.865	16.784	15.548	14.233	16.865	19.543	12.858	14.430
	0.8	14.864	13.767	12.650	14.543	14.248	15.853	14.256	12.896
	0.9	14.930	11.875	12.675	15.674	16.674	14.346	16.542	13.875
	1.0	13.864	15.754	12.870	13.000	14.850	13.763	13.963	17.860

### 3.4 Amplification and standard curves

**Figure 3:**
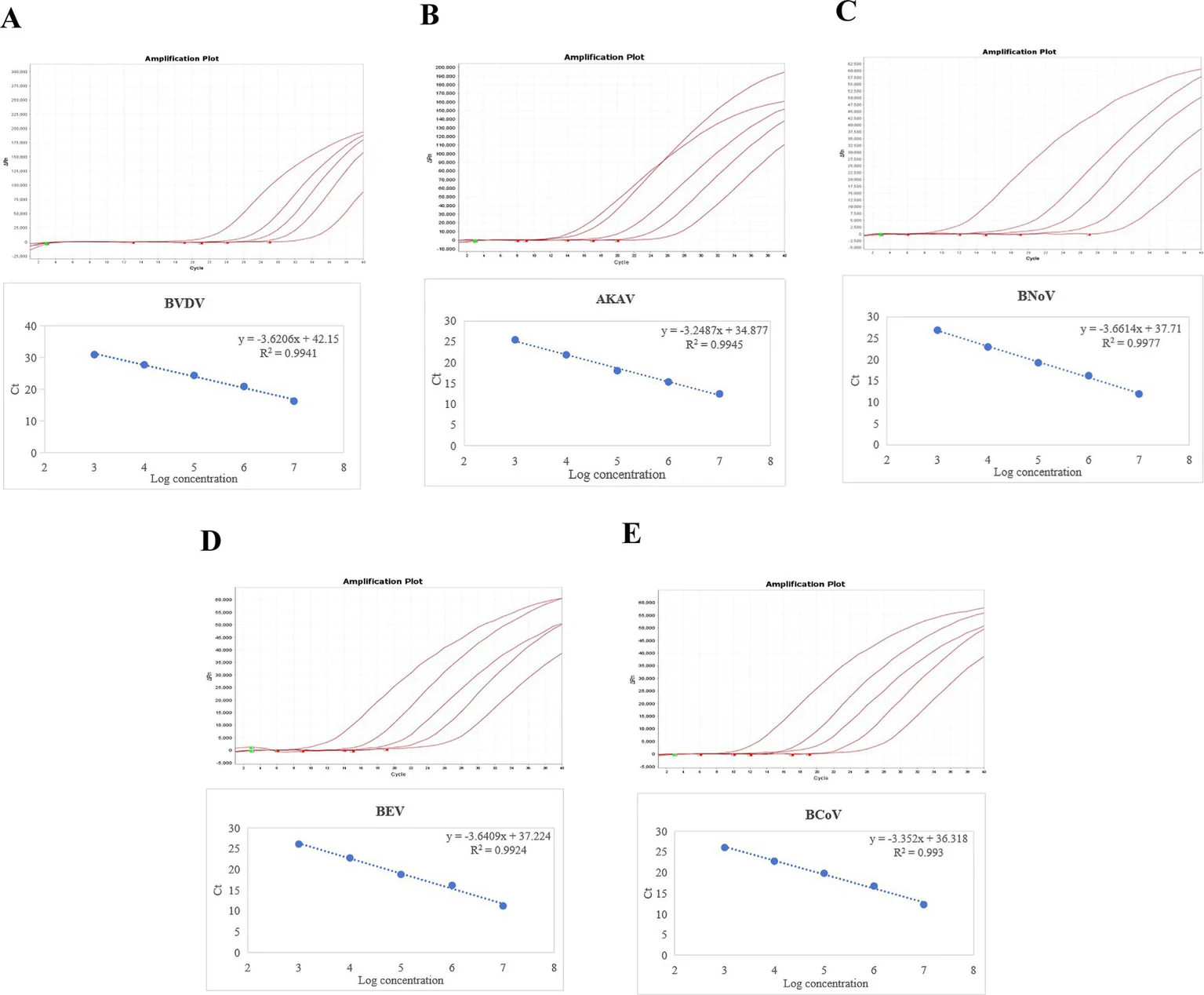
Amplification curve and standard curve. **(A)** BVDV; **(B)** AKAV; **(C)** BNoV; **(D)** BEV; **(E)** BCoV.

Multiplex real-time fluorescence qPCR amplification was performed using 10-fold serial dilutions of standard plasmids (ranging from 10^7^ to 10^3^ copies/μL) as templates. Results showed good linear relationships (Figure 3). Standard curves were as follows: for BVDV, Y = −3.6206X + 42.15, R^2^ = 0.9941; for AKAV, Y = −3.2487X + 34.877, R^2^ = 0.9945; for BNoV, Y = −3.6614X + 37.71, R^2^ = 0.9977; for BEV, Y = −3.6409X + 37.224, R^2^ = 0.9924; and for BCoV, Y = −3.352X + 36.318, R^2^ = 0.9930. The R^2^ values of all standard curves exceeded 0.9900, indicating excellent linearity.

### 3.5 Robustness analysis

**Figure 4:**
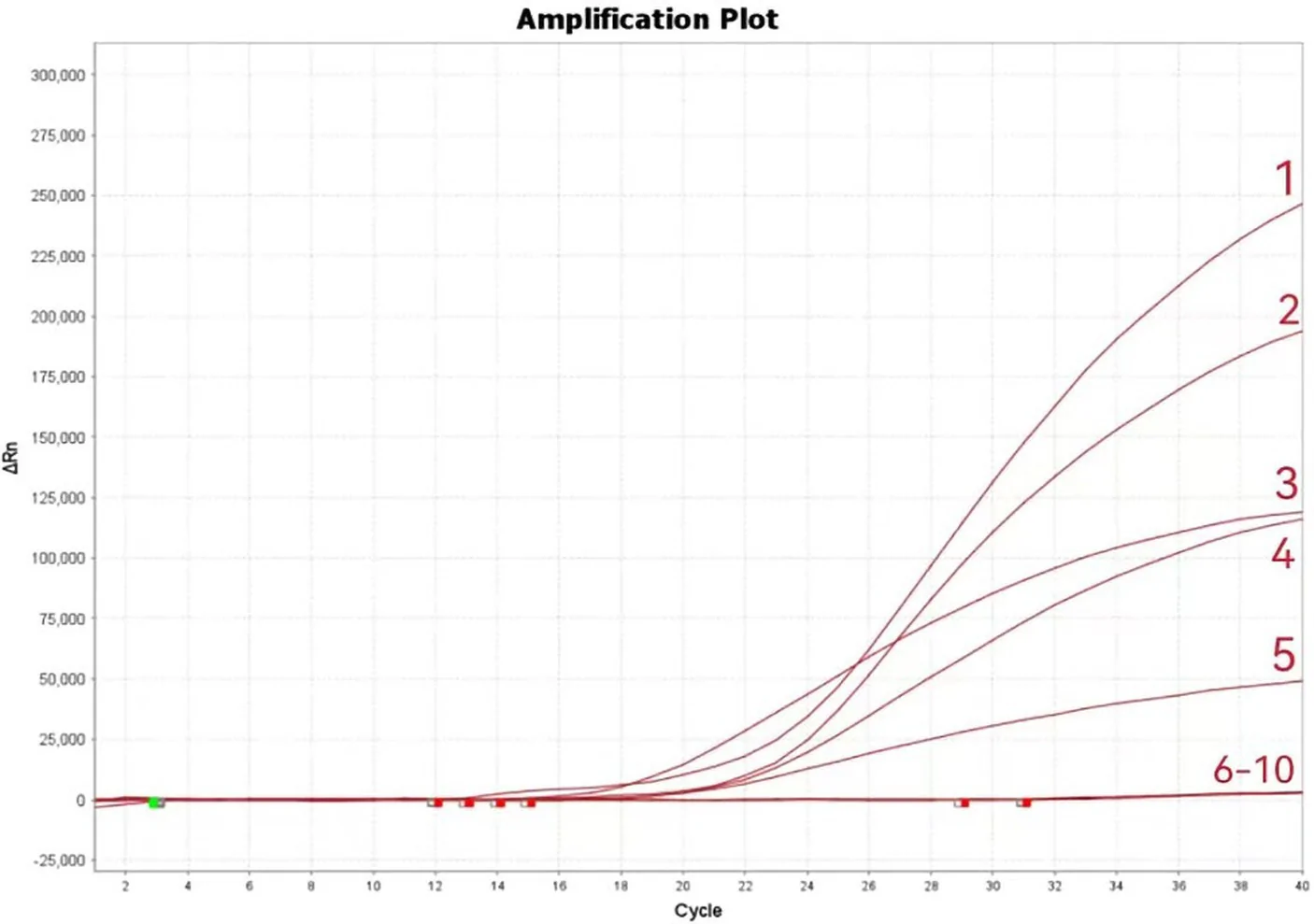
The results of anti-interference 1–5: Amplification curves of BVDV, BNoV, AKAV, BCoV, BEV; 6–10: Amplification curves of IBRV, BRV, BPV, BToV, BRSV.

The robustness of the multiplex real-time fluorescence qPCR assay was assessed by testing nucleic acid samples of IBRV, BRV, BPV, BToV, and BRSV. Results demonstrated specific amplification exclusively for the positive standard plasmids (BVDV, AKAV, BNoV, BEV, and BCoV), with no amplification curves observed for the other viral nucleic acid samples (Figure 4). These findings indicate that the developed TaqMan multiplex real-time fluorescence qPCR assay exhibits excellent anti-interference capability.

### 3.6 Specificity analysis

**Figure 5:**
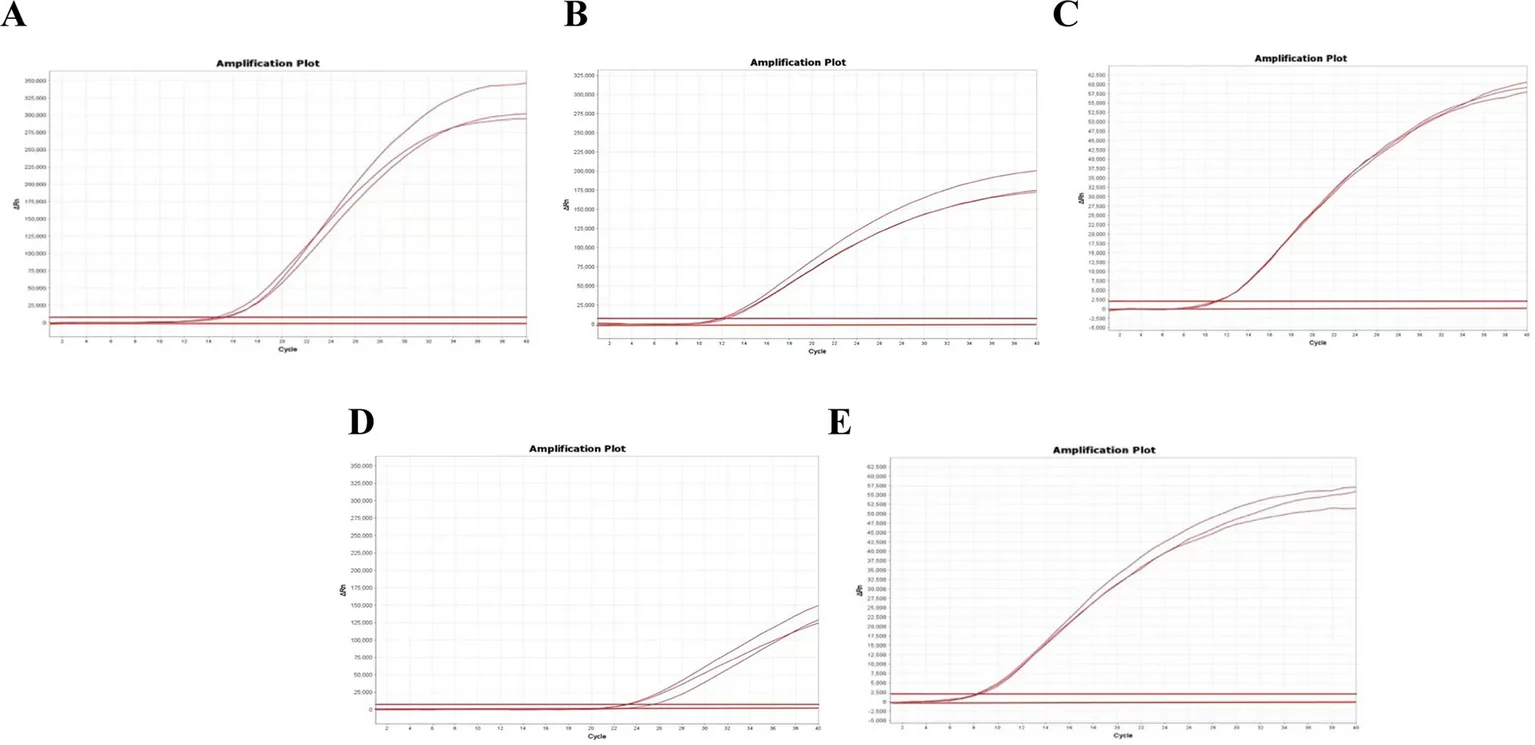
Validation of specificity. **(A)** BVDV; **(B)** AKAV; **(C)**: BNoV; **(D)**: BEV; **(E)** BCoV.

To evaluate assay specificity, cDNA templates of BVDV, AKAV, BNoV, BEV, and BCoV were amplified using the optimized reaction system in parallel with cDNA templates of IBRV, BRV, BPV, BToV, and BRSV. Each assay was performed in triplicate. Results revealed specific amplification curves only for the five target viruses, all with Ct values below 35. No amplification curves were detected for the other viruses or negative control wells (Figure 5). These results indicate that the established TaqMan multiplex real-time fluorescence qPCR assay possesses high specificity.

### 3.7 Sensitivity analysis

**Figure 6:**
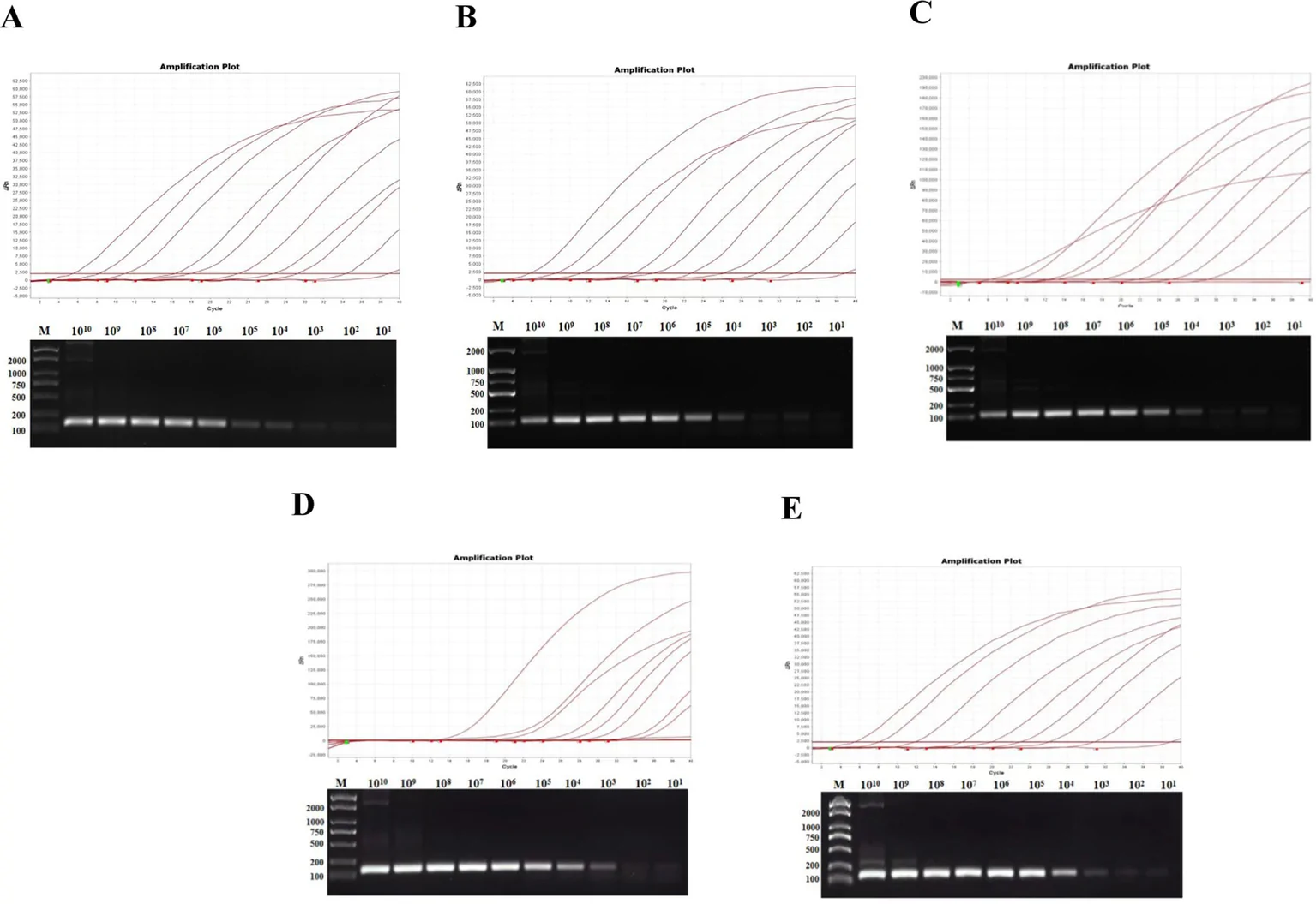
Sensitivity comparison (fluorescent quantitative PCR sensitivity and PCR sensitivity). **(A)** BVDV; **(B)**: AKAV; **(C)** BNoV; **(D)** BEV; **(E)** BCoV.

To assess assay sensitivity, tenfold serial dilutions of standard plasmids for BVDV, AKAV, BNoV, BEV, and BCoV, ranging from 10^10^ to 10^1^ copies/μL, were amplified (Figure 6). Using conventional PCR, detection limits for BVDV, AKAV, BNoV, BEV, and BCoV were 3.77 × 10^4^ copies/μL, 3.88 × 10^4^ copies/μL, 5.20 × 10^3^ copies/μL, 6.10 × 10^3^ copies/μL, and 5.48 × 10^3^ copies/μL, respectively. In contrast, the real-time fluorescence qPCR assay achieved detection limits of 3.77 × 10^1^ copies/μL, 3.88 × 10^1^ copies/μL, 5.20 × 10^3^ copies/μL, 6.10 × 10^2^ copies/μL, and 5.48 × 10^2^ copies/μL, respectively, within a linear range where Ct values were below 35. These results indicate that the real-time qPCR method exhibits significantly higher sensitivity compared to conventional PCR.

### 3.8 Repeatability analysis

To evaluate the repeatability of the assay, intra- and inter-assay tests were performed using 10-fold serial dilutions of standard plasmids of BVDV, AKAV, BNoV, BEV, and BCoV, with concentration gradients ranging from 10^6^ copies/μL to 10^4^ copies/μL. The results are presented in Table 7, where the coefficients of variation (CVs) for all groups were below 2%. These findings indicate that the established TaqMan multiplex qPCR method exhibits excellent repeatability and stability.

**Table 7:** Multiple fluorescence quantitative PCR reproducibility (CV=SD/Ct; CV < 2% represented excellent repeatability).

Samples	Copies	Inter-assay			Intra-assay		
		Ct	SD	CV/%	Ct	SD	CV/%
BVDV	3.77 × 10^6^	23.84	0.03	0.12	25.76	0.05	0.19
	3.77 × 10^5^	24.68	0.12	0.48	24.37	0.15	0.60
	3.77 × 10^4^	27.89	0.06	0.21	28.86	0.11	0.38
AKAV	3.88 × 10^6^	23.85	0.16	0.67	25.52	0.09	0.35
	3.88 × 10^5^	24.33	0.09	0.36	24.88	0.12	0.48
	3.88 × 10^4^	26.21	0.11	0.41	25.92	0.05	0.19
BNoV	5.20 × 10^6^	20.13	0.21	1.04	21.42	0.31	1.44
	5.20 × 10^5^	22.69	0.12	0.52	22.90	0.12	0.52
	5.20 × 10^4^	25.87	0.14	0.54	24.78	0.14	0.56
BEV	6.10 × 10^6^	19.68	0.08	0.41	20.66	0.09	0.44
	6.10 × 10^5^	20.12	0.22	1.09	23.58	0.14	0.59
	6.10 × 10^4^	21.86	0.13	0.59	23.89	0.25	1.05
BCoV	5.48 × 10^6^	18.36	0.06	0.32	18.97	0.21	1.10
	5.48 × 10^5^	21.88	0.04	0.18	21.06	0.11	0.52
	5.48 × 10^4^	22.75	0.12	0.52	22.23	0.09	0.40

### 3.9 Sample testing and comparative analysis

**Figure 7:**
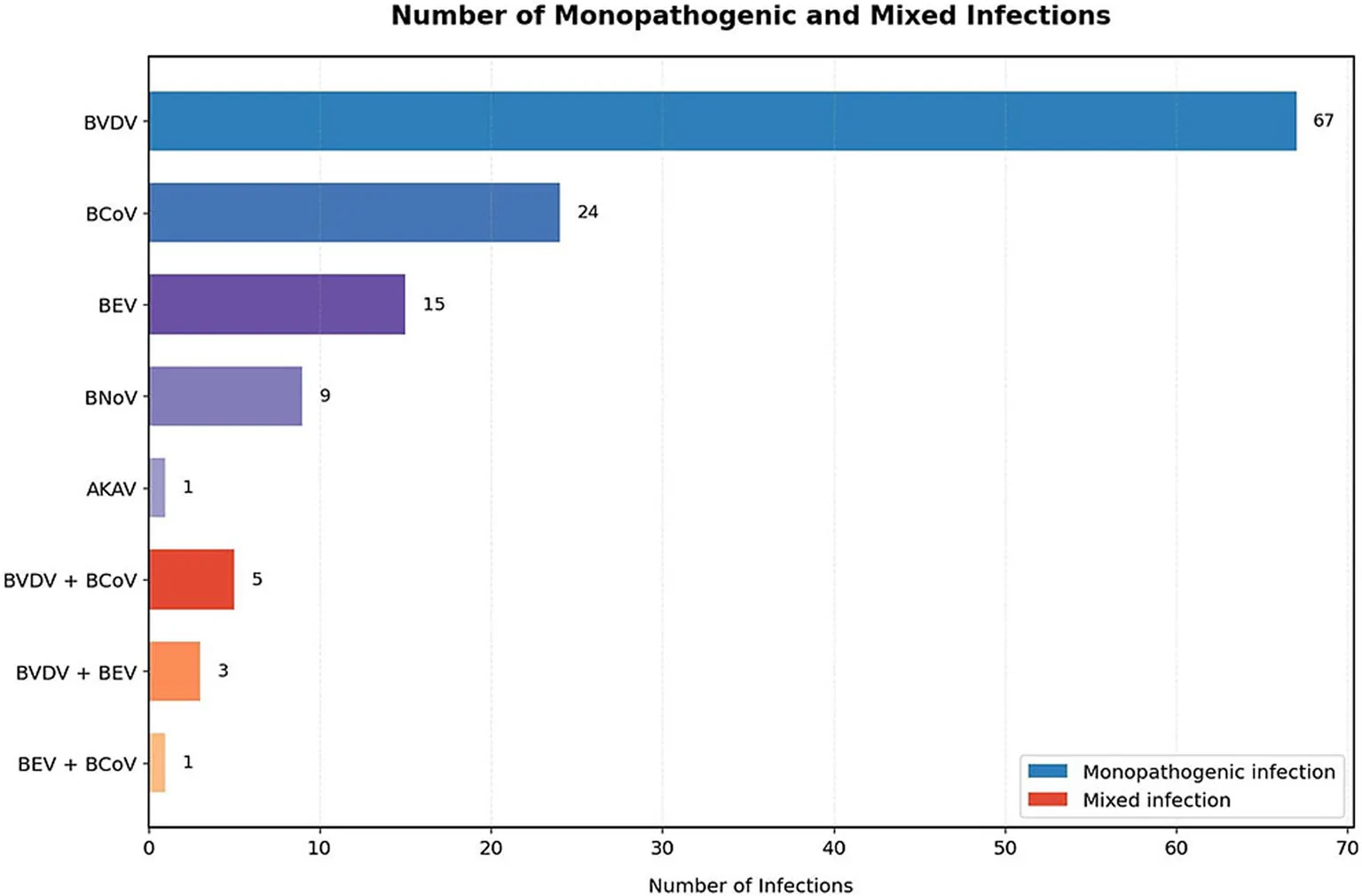
Number of single and mixed infections.

Using the established multiplex qPCR assay, a total of 200 samples were tested (20 samples were collected from each of the following locations in Jilin Province: Yanji, Siping, Dunhua, Dehui, Yushu, Gongzhuling, Baicheng, Nong’an, Huinan, and Liuhe), and the results were compared with those obtained by conventional PCR. The multiplex qPCR assay detected 67 samples as positive for BVDV, 1 for AKAV, 9 for BNoV, 15 for BEV, and 24 for BCoV. Mixed infections were identified in 3 sample with BVDV + BEV, 5 samples with BVDV + BCoV, and 1 sample with BEV + BCoV (Figure 7). The results of conventional PCR were consistent with those of the multiplex qPCR assay samples were detected. The concordance rates were 100% for BVDV, AKAV, BNoV, BEV, and BCoV.

## 4 Discussion

In its early stages, polymerase chain reaction (PCR) technology was mainly used in basic scientific research, including gene fragment cloning, mutation detection, and genome structure analysis (27). As a fundamental technique for early disease diagnosis, conventional PCR enables exponential amplification of target genes, and results are typically analyzed by agarose gel electrophoresis. However, this method has several limitations, including a high risk of false positives, band smearing, and aerosol contamination, all of which may reduce detection accuracy (28).

With advances in molecular diagnostics, real-time quantitative PCR (qPCR) has been developed. Due to its high sensitivity, specificity, and quantitative capability, qPCR is widely used in medical diagnostics and life science research (29). It is particularly useful for pathogen detection, genetic disease screening, and tumor molecular classification. The principle of qPCR involves the use of fluorescent reporters, such as SYBR Green I or TaqMan probes, to monitor fluorescence signals during amplification in real time (30). Amplification curves are generated, allowing accurate quantification of the initial nucleic acid template.

In recent years, several multiplex qPCR assays for detecting bovine pathogens have been reported. Chen et al. (31) developed a multiplex PCR assay for simultaneous detection of BVDV, bovine astrovirus (BAstV), and BRV, with limits of detection (LODs) of 5.1 × 10^2^ copies/μL, 2.57 × 10^6^ copies/μL, and 1.78 × 10^3^ copies/μL, respectively. Bao et al. (32) established a multiplex PCR system targeting BVDV, IBRV, and AKAV, achieving LODs of 10^3^ copies/μL, 10^3^ copies/μL, and 10^4^ copies/μL, respectively. Gao et al. (33) further optimized a multiplex qPCR assay for simultaneous detection of BVDV, BCoV, BRV, and malignant catarrhal fever virus (MCFV), with LODs of 9.4 × 10^1^ copies/μL, 1.4 × 10^3^ copies/μL, 9.1 × 10^1^ copies/μL, and 1.2 × 10^2^ copies/μL, respectively.

In this study, we successfully developed a TaqMan-based multiplex qPCR assay for simultaneous detection of five bovine pathogens: BVDV, AKAV, BNoV, BEV, and BCoV. Standard curves were established to systematically evaluate specificity, sensitivity, and repeatability. The LODs were 3.77 × 10^1^ copies/μL (BVDV), 3.88 × 10^1^ copies/μL (AKAV), 5.20 × 10^3^ copies/μL (BNoV), 6.10 × 10^2^ copies/μL (BEV), and 5.48 × 10^2^ copies/μL (BCoV), significantly more sensitive than conventional PCR. Both intra-assay and inter-assay CVs were below 2%, indicating excellent repeatability and stability, meeting requirements for rapid clinical testing.

Epidemiological investigations have revealed widespread prevalence of these pathogens in China. Dong et al. surveyed clinical samples from five regions in Jilin Province, reporting infection rates of 21.68% (BVDV), 14.23% (BEV), and mixed infection rates of 7.99% (BVDV and BEV) (2). Xu et al. examined cattle herds with abortion and fetal malformations in parts of Jilin Province, identifying single and mixed infections of BVDV and IBRV. Although AKAV has not yet been detected in Jilin Province, cattle abortion and fetal malformation cases in the eastern region are closely linked to AKAV infection (34). Sun et al. demonstrated the presence of BCoV across eastern, central, and western regions of Jilin Province, with positivity rates highest in the eastern region and lowest in the central region. The overall infection rate of BCoV was 66.67%, with the highest seasonal prevalence in spring (32.14%) (1). Liu et al. reported higher seropositivity rates for BVDV in areas bordering Liaoning Province (Siping City, Lishu County, Dongfeng County, and Meihekou City). In Jilin City and Fengman District, average seropositivity for BVDV was 62.52%, with antigen positivity at 19.21% (35). Liu et al. tested 326 fecal samples suspected of BEV infection from different regions of Jilin Province (2012–2016), revealing infection rates ranging from 10.5 to 20.4% (35).

In the present study, the established TaqMan-based multiplex real-time PCR assay demonstrated favorable specificity, sensitivity, and repeatability in analytical validation. When applied to 200 clinical samples collected from cattle farms in Jilin Province, the assay revealed detection rates of 33.50% for BVDV, 0.50% for AKAV, 4.50% for BNoV, 7.50% for BEV, and 12.00% for BCoV, with mixed infections identified in 9 samples (4.50%). To our knowledge, this study reports for the first time the development of a multiplex qPCR assay capable of simultaneously detecting these five bovine viruses, providing a rapid and efficient tool for differential diagnosis and epidemiological surveillance.

Among the five targeted viruses, AKAV exhibited the lowest detection rate, with only one positive case identified among 200 samples (0.5%). This result is closely associated with the ecological, epidemiological, and transmission characteristics of AKAV. First, AKAV is transmitted primarily by blood-feeding arthropods, and its transmission efficiency is substantially constrained by seasonal and environmental factors (36). Jilin Province experiences a prolonged cold season, which shortens the period of vector activity and thereby reduces transmission efficiency, resulting in a lower probability of infection compared to directly transmitted viruses (37). Second, AKAV infection is predominantly subclinical or asymptomatic, with clinical manifestations rarely observed and mainly limited to reproductive disorders in pregnant cattle (37). Since the clinical samples in this study were not specifically collected from pregnant herds, the detection rate was consequently low. Third, serological surveys have revealed a relatively high prevalence of AKAV antibodies in cattle populations in Jilin Province. Calves that acquire subclinical infection early in life develop immunological protection, which effectively reduces the likelihood of secondary infection and clinical disease. Fourth, as an arthropod-borne virus, AKAV transmission tends to be sporadic and focal, making sustained transmission chains difficult to maintain (36). Given the fixed sampling sites and time period in this study, the probability of capturing low-frequency cases was inherently limited. Collectively, the low detection rate of AKAV in this study can be attributed to a combination of transmission constraints, distinct pathogenic characteristics, herd immunity, and epidemiological patterns. Although clinical disease caused by AKAV is infrequent, subclinical infections can still lead to reproductive disorders (38); therefore, AKAV should remain incorporated into routine surveillance programs to ensure comprehensive prevention and control of relevant diseases in cattle populations.

It is noteworthy that among the 200 clinical samples tested, only dual co-infections were detected, with no triple or higher-order co-infections involving the five targeted viruses identified. This observation warrants careful consideration regarding the rationale for designing a multiplex assay. While the current clinical samples predominantly exhibited dual co-infections, the value of the multiplex format extends beyond matching the complexity of infections observed in a single cross-sectional sampling. The design of the multiplex assay was primarily based on region-specific epidemiological evidence, which demonstrated that all five viruses are prevalent in Jilin Province and frequently co-circulate in cattle populations. The strength of a multiplex detection system lies in its ability to simultaneously screen for a broader panel of pathogens in a single reaction, thereby enhancing the probability of detecting rare triple or quadruple co-infections that might otherwise be overlooked if multiple single-plex tests were performed separately.

In addition, the multiplex assay offers substantial practical advantages. By simultaneously detecting five major viral pathogens in a single reaction, it significantly reduces detection costs and turnaround time compared to single-plex assays or conventional PCR methods. This feature makes the assay particularly suitable for resource-limited settings, including large-scale farms with limited infrastructure, grassroots veterinary stations, and port quarantine facilities requiring rapid on-site screening (39). The ability to rapidly rule out or confirm infection by multiple pathogens facilitates timely implementation of targeted control measures, thereby mitigating economic losses and supporting disease surveillance efforts (40).

It is also important to recognize that pathogen circulation patterns are dynamic and may shift over time due to changes in climate, husbandry practices, animal movements, or viral evolution (41). A multiplex assay established now provides a future-ready technical platform that can promptly accommodate potential changes in co-infection complexity without requiring additional assay development (42). Therefore, although the present clinical samples showed only dual co-infections, the multiplex assay should be viewed as a proactive diagnostic tool designed not only to address current clinical needs but also to serve as a technological reserve for future epidemiological scenarios where more complex co-infections may emerge. Admittedly, this forward-looking design entails that the assay’s capacity to resolve higher-order co-infections warrants further validation with a larger and more diverse sample set collected across different seasons and geographical regions in future studies.

## 5 Conclusion

This study is the first to establish a TaqMan-based multiplex real-time qPCR assay for the simultaneous detection of five bovine viruses (BVDV, AKAV, BNoV, BEV, and BCoV) and validated its specificity, sensitivity, and repeatability. The limits of detection for these viruses were 3.77 × 10^1^ copies/μL for BVDV, 3.88 × 10^1^ copies/μL for AKAV, 5.20 × 10^3^ copies/μL for BNoV, 6.10 × 10^2^ copies/μL for BEV, and 5.48 × 10^2^ copies/μL for BCoV, demonstrating significantly higher sensitivity compared to conventional PCR methods. Notably, BVDV and BCoV showed relatively higher detection frequencies among the tested pathogens, suggesting their potentially higher prevalence in the surveyed cattle population. This method provides a reliable technical approach for the rapid differential diagnosis and epidemiological investigation of these five bovine viruses.

## Data Availability

The original contributions presented in the study are included in the article/supplementary material, further inquiries can be directed to the corresponding author.
